# A simplified immunoprecipitation method for quantitatively measuring antibody responses in clinical sera samples by using mammalian-produced *Renilla *luciferase-antigen fusion proteins

**DOI:** 10.1186/1472-6750-5-22

**Published:** 2005-08-18

**Authors:** Peter D Burbelo, Radoslav Goldman, Thomas L Mattson

**Affiliations:** 1 Lombardi Comprehensive Cancer Center, Department of Oncology, Georgetown University Medical Center, Washington, D.C. 20057

## Abstract

**Background:**

Assays detecting human antigen-specific antibodies are medically useful. However, the usefulness of existing simple immunoassay formats is limited by technical considerations such as sera antibodies to contaminants in insufficiently pure antigen, a problem likely exacerbated when antigen panels are screened to obtain clinically useful data.

**Results:**

We developed a novel and simple immunoprecipitation technology for identifying clinical sera containing antigen-specific antibodies and for generating quantitative antibody response profiles. This method is based on fusing protein antigens to an enzyme reporter, *Renilla *luciferase (Ruc), and expressing these fusions in mammalian cells, where mammalian-specific post-translational modifications can be added. After mixing crude extracts, sera and protein A/G beads together and incubating, during which the Ruc-antigen fusion become immobilized on the A/G beads, antigen-specific antibody is quantitated by washing the beads and adding coelenterazine substrate and measuring light production.

We have characterized this technology with sera from patients having three different types of cancers. We show that 20–85% of these sera contain significant titers of antibodies against at least one of five frequently mutated and/or overexpressed tumor-associated proteins. Five of six colon cancer sera tested gave responses that were statistically significantly greater than the average plus three standard deviations of 10 control sera. The results of competition experiments, preincubating positive sera with unmodified *E. coli*-produced antigens, varied dramatically.

**Conclusion:**

This technology has several advantages over current quantitative immunoassays including its relative simplicity, its avoidance of problems associated with *E. coli*-produced antigens and its use of antigens that can carry mammalian or disease-specific post-translational modifications. This assay should be generally useful for analyzing sera for antibodies recognizing any protein or its post-translational modifications.

## Background

Although it is clear that a normal host immune system recognizes and responds to tumors, we understand very little about these complex tumor-host interactions. For example, it is not clear why tumor-associated proteins elicit humoral responses, although it is often speculated that such proteins can become antigenic when they are overexpressed or represent an unusual or modified form of a protein (e.g. altered spliced form), or are encoded by mutant genes [1,2]. Efforts to identify antibody responses to tumor antigens are motivated primarily by their diagnostic potential. Unfortunately, the immunoassay formats available to most laboratories are less than ideal.

Most immunoassays use bacterial-expressed proteins for detecting antigen-specific antibodies in human sera [2]. However, since such antigens do not carry post-translational modifications or may fold incorrectly, some immunoassays employ antigens produced in either yeast or insect cells. While these antigens may fold correctly and carry post-translational modifications, they will not carry either mammalian- or disease-specific posttranslational modifications. Tests employing bacterial-produced proteins can produce high backgrounds because it is difficult to completely eliminate or block serum antibodies reactive with trace amounts of bacterial contaminants present in most antigen preparations, even in pharmaceutical grade preparations [3]. Therefore to overcome the biological limitations and technical problems associated with bacterially and non-mammalian-produced antigens, we have developed a simple immunoassay that combines conventional immunoprecipitation techniques with a novel approach for the production of tumor antigens. The tumor antigens are fused to an enzymatic reporter, Ruc, and produced in mammalian cell cultures, where mammalian-specific post-translational modifications can be added. This technology is based on our previously published studies showing that a Ruc fusion with a human protein retain the biological activities of both the reporter and the human protein and can be used to detect weak protein-protein interactions [4]. In the present application, we utilized such fusions to detect protein-antibody interactions.

Our immediate interest in this technology is that we believe it can be used to systematically test the hypothesis that, in sporadic cancers, mutant or overexpressed tumor-associated proteins frequently induce humoral responses. While variations of this hypothesis have be proposed, it has not been vigorously tested for mainly historical and technological reasons. Until recently, only a few frequently mutated tumor-associated proteins were known [5]. For example, in sporadic breast and colon cancers, mutations in only a few proteins, p53 and SMAD4 or p53, k-Ras and APC, respectively, had been identified prior to 2001. Recent molecular genetic studies have greatly increased the number of genes known to be mutant in different types of sporadic cancers. For example, in colon cancers over 15 different genes are known to be frequently mutated [6-10], although individual patient tumors are highly heterogeneous in their mutant gene spectrum. In light of the fact that accurate classification of patient tumors into well-defined subtypes by gene expression profiling requires a panel of genes, each of which may be specifically up- or down-regulated in only a small percentage of tumors [11-15], we hypothesize that monitoring humoral immune responses to a panel of frequently mutated and/or overexpressed tumor-associated proteins in cancer patient sera can be used in an analogous manner, but with the added advantage of not requiring tumor tissue. In addition, existing data suggests that cancer patients' antibody responses to these mutant proteins are generally not limited to the mutated region of the protein. For example, colon cancer patient sera containing anti-Ras antibodies were equally reactive with either wild type or mutant K-Ras recombinant proteins [16]. Epitope mapping experiments showed that these sera always reacted with the C-terminus of K-Ras, although the mutated amino acid is almost always at the N-terminus of Ras. Similar results studying antibody responses to p53 were obtained with sera from patients with breast, colorectal and lung cancer [17]. While p53 mutations are in the central region, the majority of immunodominant epitopes are in the N- and/or C-termini of p53 [18,19]. For both mutant CDX2 in colon cancer, and mutant B-Raf in melanoma, patient antibodies react with both the wild type protein and mutant epitopes [20,21]. Here we describe a simple practical quantitative immunoprecipitation assay that has a number of practical advantages including that it is inexpensive, easy-to-perform and can be used for detecting antigen-specific antibodies in clinical sera samples. The proteins used here, as antigens are frequently mutated or overexpressed in the types of tumors carried by the patients whose sera are used to demonstrate the usefulness of this new immunoassay format.

## Results and discussion

### Description of the immunoprecipitation assay

We used Ruc-tagged proteins to develop an immunoprecipitation assay that can quantitatively measure serum antibody reactivity with protein antigens. Briefly, crude extract containing the Ruc-antigen fusions, sera and protein A/G beads are mixed together and incubated, during which the antigen fusions become immobilized; antigen-specific antibody is then quantitated by washing the beads and adding the colenterazine substrate. In these assays the amount of light produced is proportional to the amount of soluble fusion protein captured, directly or indirectly by the antibody-bound beads. It should be noted that the binding capacity of the protein A/G beads (Pierce Biochemical) used to capture either purified monoclonal antibodies or immunoglobulins from crude human or animal antisera is quite high (24 μg of immunoglobulins/μl of packed beads).

### The immunoprecipitation assay shows a linear range of detection with commercial antibodies

**Figure 1:**
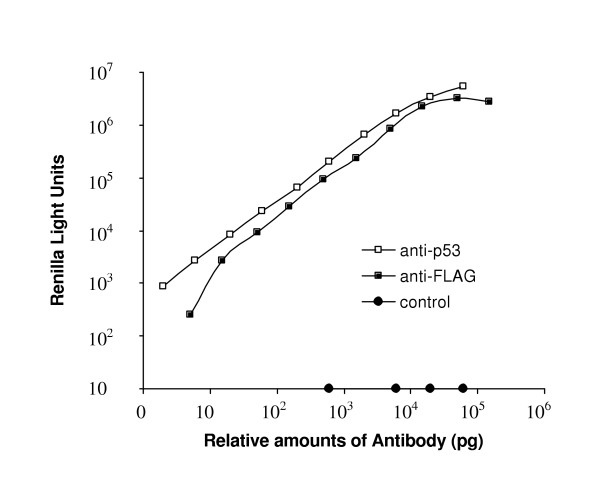
Immunoprecipitation experiments with commercial antibodies. Various amounts of anti-FLAG monoclonal, anti-p53 polyclonal or control (anti-WASP) polyclonal antibodies were mixed with 5 μl of a Cos1 extract containing Ruc-p53 for 1 h in the presence of protein A/G beads, processed and light units measured. The data shown is from one of three independent experiments giving similar results.

To illustrate this technology we generated Ruc fusion protein constructs for p53, K-Ras, c-Myc, β-catenin and Smad4 by fusing cDNAs encoding these proteins (in frame) to DNA encoding the C-terminus of Ruc in a mammalian expression vector, pREN2, which also encodes a FLAG epitope tag at the N-terminus of Ruc. Transfections into Cos1 cells of these different constructs yielded crude extracts with 3–10 × 10^8 ^Ruc light units per 100 mm^2 ^plate. We developed a standard assay format by using a commercial anti-FLAG monoclonal antibody and Cos1 cell extracts containing Ruc-p53. When crude extract, antisera and protein A/G beads were incubated together in a single tube, the amount of immunoprecipitated Ruc-p53 was directly proportional to the amount of anti-FLAG antibody over a 1000-fold range of concentrations, with a lower limit of detection of less than 5 picrograms (Figure 1). A commercial anti-p53 polyclonal antibody had a similar capture capacity as reflected by a similar dose-response curve, whereas a commercial polyclonal antibody against an unrelated antigen was unable to immunoprecipitate Ruc-p53 (Figure 1). Experiments using commercial polyclonal antibodies for serine-15 phosphorylated p53 and acetylated p53 (lysine-373 and lysine-382) also immunoprecipitated significant amounts of Ruc-p53 (data not shown). Since the ability of these modification-specific antibodies to immunoprecipitated Ruc-p53 was not competed by bacterially produced recombinant p53 protein (data not shown), this fusion protein appears to contain at least two types of post-translational modifications.

### Human cancer patient sera contain antigen-specific antibodies

Since commercial antibodies can immunoprecipitate Ruc-antigen fusions from crude Cos1 extracts, we tested whether our simple assay format could also detect antigen-specific antibodies in clinical sera samples. Our motive for developing this technique was to have an improved method for detecting cancer patient antibody responses to tumor-associated proteins. Thus, we initially tested it with a small number of clinical sera samples taken from patients having three types of cancers, breast, colon and head and neck. In order to maximize our chances of detecting positive responses with these clinical sera samples we chose to use p53 and four other tumor-associated proteins (K-Ras, c-Myc, β-catenin and Smad4) that are either frequently mutated and/or overexpressed in various tumors. Wild type proteins were used as antigens because several studies show that cancer patient sera humoral immune responses are not restricted to or even preferential for the epitopes that usually contain the altered amino acids [16,18-21]. Cos1 extracts containing Ruc-antigen fusions were used to test a total of 36 sera, 10 controls and 26 cancer patients (Table 1). Negative and positive controls consisting of protein A/G beads alone and 0.1 μg of anti-FLAG monoclonal antibody with protein A/G beads, respectively, were used for each experiment. As expected, all sera had low reactivity with the non-specific binding control protein, Ruc-alone (Table 1). The positive control, anti-FLAG antibody, immunoprecipitated significant amounts of each of the Ruc-antigen fusions. However, the fraction of the total Ruc activity that could be captured varied amongst the different Ruc-antigen fusions, possibly reflecting reduced accessibility to the N-terminal FLAG epitope in some constructs (data not shown). At least one cancer patient sera had statistically significant antibody responses to each of the five Ruc fusions, where significance is defined as a response greater than the average plus three standard deviations of the 10 control sera (Table 1). Two of 10 head and neck, five of 10 breast, five of six colon cancer sera, but none of 10 healthy control sera gave positive responses. Six of the 12 positive tests were clustered in the six colon cancer patient sera and two antigens, p53 and K-Ras (Table 1). The significance of the relative response rates between different cancer-type sera cannot be calculated because the sample sizes are small and because no effort was made to match the control and patient sera by any criteria. Similarly, we cannot conclude that either K-Ras and/or p53 may be more antigenic in colon cancers than either β-catenin or c-Myc. Interestingly, the only multiple sample from any of the patients, head and neck samples 11 and 12, are sequential samples of which only the more recent sample showed significant levels of anti-p53 antibodies. Since the proteins used to test for antibodies in these 26 cancer patient sera are often mutated and/or overexpressed in the three types of cancer, our results are consistent with studies indicating that these categories of proteins are often antigenic in cancer patients [2]. Our results with colon cancer patient sera also support proposals that humoral immune responses to panels of tumor-associated antigens may be clinically useful when single antibody responses are not informative [22,23]. In any case, the detection of cancer sera from head and neck (20% sensitivity and 100% specificity), breast (40% sensitivity and 100% specificity), and colon (86% sensitivity and 100% specificity) obtained using a panel of five antigens are encouraging, given that our assay is not yet optimized and the sample sizes are small.

**Figure 2:**
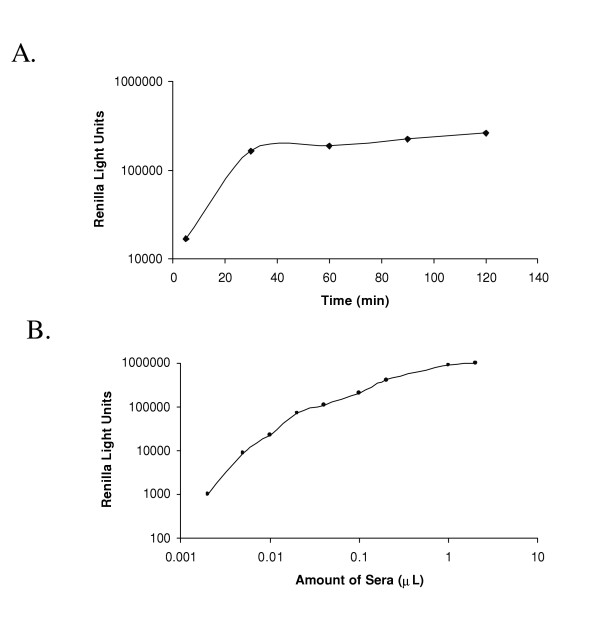
The immunoprecipitation assay with Ruc-p53 and a clinical serum sample. A: The immunoprecipitation activity is proportional to incubation time. Tubes containing identical amounts of Ruc-p53 fusion protein extract (5 μl), patient 34 sera (1 μl) and protein A/G beads were incubated for 5, 30, 60, 90 and 120 min and processed for luciferase activity. B: Immunoprecipitation activity with various amounts of total crude patient 34 sera. Different amounts of patient sera (0.002 to 2 μl) were mixed with 5 μl of the Ruc-p53 fusion protein extract and incubated for 1 hour in the presence of protein A/G beads, processed and light units measured. The data shown is from one of three independent experiments giving similar results.

To determine whether patient antibody responses behave in the same linear manner as the commercial antibodies, we used the most reactive combination of patient sera and fusion antigen in our small sample set, colon cancer sera 34 and the Ruc-p53 fusion. Although the amount of Ruc-p53 captured by this serum is roughly linear with incubation time in the presence of protein A/G beads, reaching a plateau by 30–60 minutes (Figure 2A), the relative amount of immunoprecipitated Ruc-p53 was not completely linear with increasing amounts of sera (Figure 2B). Since the two commercial antibodies used in Figure 1 are highly purified, the non-linear dose-response curve of the clinical sera sample could be due to interfering agents such as anti-p53-specific IgA and IgM antibodies that recognize epitopes also recognized by IgG's but which bind poorly to protein A/G beads [24]. We are exploring modifications of the assay format in order to produce a more linear dose-response curve with clinical sera samples, which if found, would facilitate assay standardization and might increase sensitivity (note that 1–2 of the colon cancer responses to one of the antigens tested, β-catenin-Δ1, are barely below the cut-off value used to judge statistical significance). If the presumed interfering agents also affected ELISA tests, ELISA tests may significantly underestimate positive antibody responses and antibody titers unless the sera are sufficiently diluted.

#### Competition experiments with unmodified proteins

**Figure 3:**
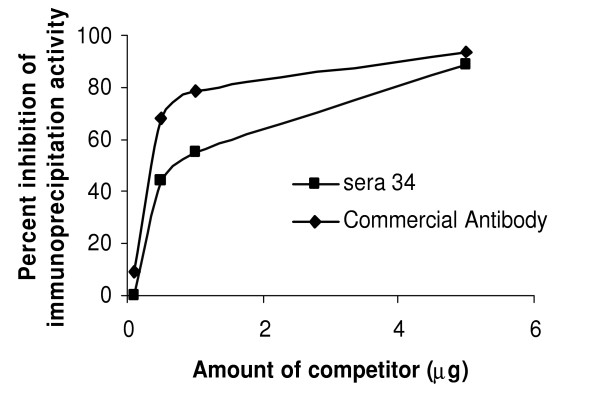
Competition assays blocking Ruc-p53 immunoprecipitation using bacterially-produced antigen. Different amounts of *E. coli*-produced MBP-p53 were incubated with patient sera 34 (0.5 μl) or commercial anti-p53 antibody (25 ng) for 1 h. Protein A/G beads and Ruc-p53 extract were then added and incubated for an additional 1 h, processed and light units measured. The data shown is from one of two independent experiments giving similar results.

While human humoral immune responses to post-translational modifications are often ignored and/or undetectable with existing technologies, recent studies demonstrate that disease-related antibody responses can occur to post-translational protein modifications [25]. In at least one case, rheumatoid arthritis, antibody responses to a post-translational modification, citrullination, is now being intensely investigated as a potentially reliable disease indicator [26,27]. In light of these observations, we asked whether each positive sera response seen in Table 1 could include antibodies that were directed toward post-translational modifications by doing competition experiments with unmodified *E. coli*-produced antigens. These competition experiments (Table 2) show that 0–100% of the immunoprecipitated Ruc-antigen fusions were blocked by preincubating sera with 5 μg of the corresponding *E. coli*-produced antigens fused to maltose binding protein (MBP). These differences occur even between sera containing antibodies that recognize the same antigen (e.g. p53 or K-Ras), proteins known to contain post-translational modifications. These differences could mean that some tumors tend to produce proteins having more post-translational modifications or that some cancer patient's immune system tend to produce significantly more antibodies that recognize post-translational modifications. However, this data does not exclude the possibility that some or all of each positive antibody response detected is not even specific for the antigen listed, since the apparent anti-p53 or anti-K-Ras antibodies could be directed toward proteins that are in complexes with these tumor antigens. If the tumor antigens in these complexes were easily replaced by the MBP fusions, one would see higher competition values than if they were inefficiently replaced. Quantitative evaluation of different competition results requires, at a minimum, equal amount of reactive antibodies in each sera, a condition unlikely to be satisfied here, especially for the p53-reactive sera. In addition, when we compared the dose-response competition curves of sera 34 and the commercial polyclonal anti-p53, adjusted to similar capacities for immunoprecipitating Ruc-p53, we found a greater difference than indicated by the end-point values alone (Figure 3). Nevertheless, it is clear that our assay identifies patient sera having qualitatively different humoral immune responses to the same antigen. Additional tools, especially antigens bearing only a single type of modification, will be required to determine whether some or all of the presumed "low affinity" antibodies prefer epitopes bearing post-translational modifications because many such antibodies are likely to cross-react with unmodified epitopes.

We have preliminary observations suggesting that our approach of making antigen-enzyme fusions and producing these fusions in mammalian cells may be superior to conventional ELISA assays for detecting antigen-specific antibody responses in human sera. Specifically, we have tested the six colon cancer patient sera used here in a standard sandwich type ELISA where the antigen were fused to *E. coli *MBP and immobilized on ELISA plates with a monoclonal anti-MBP antibody. In these ELISA tests only two of the six colon cancer sera gave positive responses with any of the five tumor-associated proteins listed in Table 1 (data not shown). In any case, the immunoprecipitation assay described here offers a practical approach for identifying post-translational modification-specific antibody responses and studying their medical relevance.

## Conclusion

These results demonstrate that a simple quantitative immunoprecipitation assay can identify human clinical sera samples containing disease-related antigen-specific antibodies. Quantitative results were obtained by using easily prepared crude cell extracts containing post-translationally modified antigens fused to a light-producing enzyme reporter. While the immunodetection of antigen-enzymes is not new [28,29], by combining a robust reporter, such as Ruc with the production of recombinant enzyme-antigen fusions in mammalian cells, we have created a highly sensitive user friendly assay. This assay requires fewer manipulations for reagent preparation and less time than other immunoprecipitation methods including avoiding having to purify and then radiolabel the purified proteins or having to perform additional analysis such as Western blotting after the immunoprecipitations [30]. Producing the target antigens in mammalian cells offers several potential advantages, including having mammalian-specific and/or disease-specific post-translational modifications added to these antigens. Thus, this immunoprecipitation assay provides a simple, accessible, reliable and reproducible tool for investigations aimed at documenting the role of post-translational modification in disease. Although altered post-translationally modified proteins occur in cancer [31,32], future studies are needed to explore whether there are detectable cancer patient-specific antibodies to post-translationally-modified tumor proteins. The levels and kinds of post-translational modifications on the Ruc-antigen fusions can be manipulated by exploiting mutant proteins, unique human cell lines (e.g. cell lines overexpressing tyrosine kinases) and various culture conditions. Mammalian-produced antigens have additional advantages over bacterial produced antigens including facilitating the study of antibody responses to very large proteins (>100 kDa) that are difficult or impossible to produce as intact proteins in *E. coli*. Our assay also avoids false positives caused by variable amounts of anti-*E. coli *antibodies present in patient sera that react with the minor amounts of *E. coli *proteins that co-purify with bacterial recombinant proteins; such contaminants are even present in some pharmaceutical-grade recombinant protein preparations [3]. These advantages, along with the possibility of improving the assay format, suggest that it may be worthwhile to use this assay to reevaluate the frequency with which known tumor-associated proteins are detectably antigenic in cancer patients. It is encouraging, although of limited significance, that the frequencies of significant antibody responses for two of the cancers are roughly comparable to reports in the literature. Thus, in colon cancer patients we detected statistically significant antibody responses to Ras and p53 in 50% and 33% of the sera, respectively, compared to published reports of 33% for Ras [16] and 26% for p53 [33]. In contrast, we did not find any statistically significant antibody responses to p53 in breast cancer sera, which have been reported to occur with 9% of patient sera [34]. Studies with much larger sample numbers are clearly needed to make statistically useful comparisons between our method and existing methods.

This assay format and high throughput modifications (e.g. magnetic A/G beads in a microtiter plate format) are obviously directly applicable to detecting human sera antibodies specific for any protein antigen of interest and is likely to be useful for non-human sera, such as sera obtained from animal models of disease, as well as for antibodies in other bodily fluids including from ascites and saliva. Variations of this immunoprecipitation assay format might also be useful for studying other types of protein-protein interactions.

## Methods

### Biochemical reagents and antibodies

Ultralink™ immobilized protein A/G beads were obtained from Pierce Biotechnology Inc. Commercially available antibodies were: mouse monoclonal anti-FLAG™ M2 from Sigma; rabbit anti-acetylated p53 from Upstate Biochemicals and polyclonal rabbit anti-p53, polyclonal rabbit phosphoserine p53 and polyc lonal anti-WASP from Santa Cruz Biotechnology.

### Patient sera

The breast and colon cancer patient sera were obtained from the University of Wisconsin collection, now kept at Georgetown University Medical Center. Sera samples from head and neck cancer patients and control sera were collected by Dr. Radoslav Goldman at Georgetown University Medical Center (Washington, DC). The sex, age and disease stages of these samples were not examined until after the reactivities for all antigens were measured.

### Generation of constructs encoding Ruc fused to tumor-associated antigens

**Figure 4:**
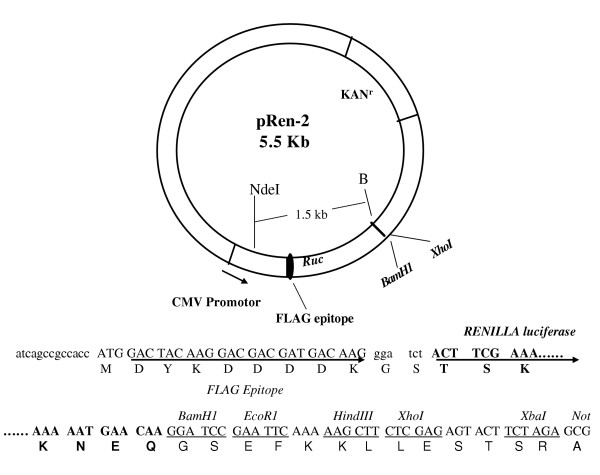
Structure of the pREN2 mammalian expression vector. Features indicated are CMV (cytomegalovirus) promoter, the N-terminal FLAG epitope and Ruc. Sequences for Ruc are in bold. cDNAs for tumor antigens were cloned downstream of Ruc between the *BamH1*-*Xho1 *sites.

pREN2, a FLAG-epitope-tagged mammalian expression vector, similar to the previously described pREN1 [4], was used to generate all plasmids encoding Ruc fusions. The tumor antigens are at the C-terminus and a single FLAG tag is at the N-terminus of Ruc. A map of pREN2 is shown in Figure 4. The cloned human cDNA fragments, amplified by PCR specific linker-primer adapters, were obtained from Dr. E. Chang (p53), Dr. R. Lechleider (Smad4), Dr. S. Byers (β-catenin), Dr. R. Dickson (c-Myc) and a publicly available cDNA clone (IMAGE ID 6714574) for K-Ras. Full-length coding sequences (excluding the initial methionine) were used for the tumor antigens, with the exception of the β-catenin, which encodes amino acids 2–277. In every case a stop codon was included after the C-terminal coding sequences of the tumor antigens. The primer adapter sequences used for cloning each antigen are as follows: p53, 5'-GAGGGATCCGAGGAGCCGCA GTCAGAT-3' and 5'-GAGCTCGAGTCAGTCTGAGTCAGGCC-3'; K-Ras, 5'-GAGGGATCCACTGAATATAAACTTGTG-3' and 5'-GAGCTCGAGTTACATAATTACACACTT; Smad4, 5'-GAGGGATCCGACAATATGTCTATTACG-3' and 5'-GAGCTCGAGTCAGTCTAAAGGTTGTGG-3'; β-catinin-Δ, 5'-GAGGGATCCGCTACTCA AGCTGATTTG-3' and 5'-GAGGTCGACTCAACCAGCTAAACGCACTGC-3'; and c-Myc, 5'-GAGG GATCCCTCAACGTTAGCTTCACC-3' and 5'-GAGCTCGAGTTACGCACAAGAGTTCCG-3'. For Ruc alone, a separate construct was prepared containing a stop codon at the end of the luciferase coding sequence in place of the polylinker present in pREN2.

### Immunoprecipitation assays with Ruc fusion proteins

Forty-eight hours after Fugene-6 transfection, Cos1 cells in 100 mm^2 ^plates were washed twice with PBS, scraped with 1.0 ml of Buffer A (20 mM Tris, pH 7.5, 150 mM NaCl, 5 mM MgCl_2_, 1% Triton X-100) plus 50% glycerol and protease inhibitors (10 μg/mL each of leupeptin, aprotinin and pepstatin), sonicated, centrifuged at 13,000 × g for 4 min, supernatants collected and used immediately or stored at -20°C. Total luciferase activity in 1 μl of each crude extract was measured by adding it to 100 μl of assay buffer and substrate mixture (*Renilla *Luciferase Reagent Kit, Promega) in a 12 × 75 mm glass tube, vortexing and immediately measuring light-forming units with a luminometer (GeneProbe) for 10 sec. Lysate prepared from each 100 mm^2 ^plate of transfected Cos1 cells typically provides enough extract for 60–200 assays. These crude Cos1 extracts containing these Ruc fusions were stable for at least a few weeks when stored in 50% glycerol at -20°C.

Immunoprecipitation assays were performed in 100 μl volumes containng 6 μl of a 30% suspension of protein A/G beads (in PBS), 1–10 μl sera (undiluted or diluted in Buffer A plus 100 μg/ml BSA), sufficient Cos1 cell extract to generate 1–5 million light units (usually 5 μl to 10 μl) and Buffer A and incubated at 4°C with tumbling for 5–120 minutes, washed 4–5 times with 1.2 ml of cold Buffer A and once with 1.0 ml of PBS. After the final wash, the beads, in a volume of about 10 μl, were added to the Ruc substrate and light units measured as described above. Since the capacity of these protein A/G is 24–32 mg/ml of packed beads, 2 μl of packed beads should be sufficient to immobilize most or all of the IgG in 1 μl of undiluted sera (assumed to be 10 mg/ml IgG). The amount of IgG in 2 μl of each sera that actually bound to protein A/G beads was estimated by measuring the amount of bead-bound sera released by a low pH glycine elution buffer and measured using the BCA Protein Assay kit (Pierce Biotechnology Inc.). The protein values varied from 2.0 μg to 7.3 μg/μl of patient sera (see Additional file 3).

Competition experiments were performed using MBP fusion proteins. Bacterial expression vectors were constructed by subcloning cDNA fragments into the pMAL-c2 vector (New England Biolabs). Recombinant MBP fusion proteins were produced in bacteria, purified by amylose-agarose affinity and eluted with maltose as described by the manufacturer and stored frozen or in 50% glycerol at -20°C. An MBP fusion containing the SPEC2 cDNA [35] was produced and used as a non-specific inhibitor. The integrity of the proteins was confirmed by SDS-PAGE electrophoresis and protein concentration determined. Diluted patient sera (10 μl used of sera diluted 1:10 in buffer A containing 100 μg/ml BSA) were used in the competition experiments described in Table 2, while only 5 μl of 1:10 diluted colon patient sera 34 was used in the experiments described in Figure 3.

## Authors' contributions

RG provided the sera samples for the controls and head and neck cancers. TLM produced the recombinant fusion proteins used for the competition experiments and assisted in reducing the concept to practice and manuscript writing. PDB* first conceived of the concept, generated the constructs and performed the immunoprecipitation experiments.

## Supplementary Material

Additional File 3 Table 3. Amount of protein (IgG) bound to A/G bead (μg/1 μl) from different sera used in this study Click here for file

Additional File 1 Table 1. Mean and standard deviations of sera reactivity Click here for file

Additional File 2 Table 2. Competition of antibody responses by unmodified antigens Click here for file

## Figures and Tables

**Table 1:** Immunoprecipitation capacity of 1 μl of human sera for Ruc-tumor antigen fusion proteins^a^

		Ruc	p53	K-Ras	Smad4	β-CAT-Δ1	c-Myc
Controls	1	194	19,319	480	10,582	269	4,752
	2	9	9,830	1,064	3,575	835	2,913
	3	8	5,236	445	1,773	211	2,006
	4	38	3,187	477	1,919	530	1,831
	5	14	11,908	795	6,884	161	3,346
	6	31	5,390	823	1,724	235	2,050
	7	76	22,526	1,909	6,996	259	11,816
	8	29	15,338	943	8,043	445	3,475
	9	10	12,282	1,162	19,380	215	3,623
	10	9	11,130	1,109	4,429	501	5,060

+ 3 *SD^b^*		*214*	*30,234*	*2,237*	*22,788*	*997*	*12,874*

Head and Neck	11	0	10,904	508	2,721	196	2,193
	12	0	**31,593**^c^	738	4,822	465	3,801
	13	0	12,367	840	1,868	673	4,407
	14	13	14,705	1,012	5,666	195	1,837
	15	33	**31,733**^c^	1,189	5,264	552	4,107
	16	121	4,828	621	980	279	1,974
	17	0	8,517	1,160	8,396	336	2,958
	18	0	19,240	1,283	9,485	327	1,814
	19	0	11,224	1,517	4,454	410	4,370
	20	28	7,322	554	2,261	723	2,343

Breast	21	44	13,211	960	10,219	308	5,988
	22	10	18,814	696	**42,970**^c^	302	5,450
	23	38	14,598	608	8,484	339	4,336
	24	77	11,587	1,655	17,297	**2363**^c^	3,431
	25	17	19,954	532	10,184	772	**15,650**^c^
	26	25	9,538	195	5,962	300	1,646
	27	10	7,815	**2,561**^c^	20,628	426	3,524
	28	21	15,607	308	7,380	284	1,579
	29	0	18,058	160	6,790	304	2,333
	30	**245**^c^	25,479	1,919	9,727	495	3,787

Colon	31	4	6,656	1,204	3,252	267	1,763
	32	40	20,928	**4,293**^c^	5,567	962	6,143
	33	42	**34,703**^c^	1,472	10,830	716	4,906
	34	51	**300,943**^c^	**6,439**^c^	2,610	992	3,789
	35	35	5,670	**3,306**^c^	3,860	477	1,772
	36	44	6,516	695	**37,344**^c^	371	2,395

^a^Sera, FLAG-Ruc-fusion extracts, protein A/G beads and buffer were mixed together, incubated for 60 minutes and processed. The data, light units, is the average of two experiments and is corrected for background (beads plus extract, but no sera). The standard deviation for each value is also available (see Additional file 1).

^b^Values of the averages of the 10 control sera plus 3 standard deviations.

^c^Numbers in bold are statistically significant: greater than the average plus 3 standard deviations of the 10 control sera.

**Table 2:** Competition of antibody responses by unmodified antigens^a^

Antigen/sera	Control	p53	K-Ras	Smad4	β-CAT-Δ1	c-Myc
p53/12	21%	32%				
p53/15	20%	60%				
p53/33	7%	88%				
p53/34	11%	72%				
K-Ras/27	5%		91%			
K-Ras/32	25%		82%			
K-Ras/34	4%		0%			
K-Ras/35	16%		100%			
Smad4/22	4%			92%		
Smad4/36	0%			93%		
β-catenin-Δ1/24	23%				96%	
c-Myc/25	0%					22%

^a^Sera (1 μl), buffer and 5 μg competitor were incubated together for 60 min before adding the fusion extracts and protein A/G beads for an additional 60 minutes and processed. Background light units (beads plus extract but no sera) were subtracted before calculating percent competition. The first column identifies the antigen-sera combination tested. The other columns give the amount of competition obtained for each competitor antigen. All competitors, including the control (SPEC2), are MBP fusion proteins. Values are the averages plus from two independent experiments. The standard deviation for each value is also available (see Additional file 2).
