# Supplementary material for: A simplified immunoprecipitation method for quantitatively measuring antibody responses in clinical sera samples by using mammalian-produced Renilla luciferase-antigen fusion proteins
Source: BMC Biotechnol. 2005 Aug 18;5:22. doi: 10.1186/1472-6750-5-22 (PMC1208859; doi:10.1186/1472-6750-5-22)
Supplement: Additional File 3 — Table 3. Amount of protein (IgG) bound to A/G bead (μg/1 μl) from different sera used in this study [file 1472-6750-5-22-S3.pdf]

Additional File 3. Amount of protein (IgG) bound to A/G bead (mg/1ml) from different sera used in this study[a]

Controls	Amount IgG (μg/1μl)[b]
1	7.3
2	4.7
3	4.5
4	7.1
5	4.7
6	5.7
7	5.6
8	6.0
9	4.3
10	5.3
Head and Neck Cancer	
11	4.7
12	2.9
13	3.7
14	2.0
15	6.4
16	5.0
17	3.5
18	2.5
19	4.1
20	2.1
Breast Cancer	
21	5.2
22	4.4
23	4.0
24	3.6
25	5.4
26	3.8
27	5.2
28	4.3
29	4.2
30	4.6
Colon	
31	3.9
32	3.7
33	2.7
34	3.3
35	2.9
36	2.7

[a]Sera (2 ml), protein A/G beads and buffer were mixed together, incubated for 60 minutes and the beads washed as described for performing the immunoprecipitation. Bound protein was eluted with 0.1 M glycine, pH 2.3. The amount of protein in the eluant was measured using the BCA Protein Assay kit (Pierce Biotechnology Inc.).
[b]Values are from a single determination.
