# Supplementary material for: A simplified immunoprecipitation method for quantitatively measuring antibody responses in clinical sera samples by using mammalian-produced Renilla luciferase-antigen fusion proteins
Source: BMC Biotechnol. 2005 Aug 18;5:22. doi: 10.1186/1472-6750-5-22 (PMC1208859; doi:10.1186/1472-6750-5-22)
Supplement: Additional File 1 — Table 1. Mean and standard deviations of sera reactivity [file 1472-6750-5-22-S1.pdf]

Table 1. Mean and Standard Deviations of Sera Reactivity[a,b]

Controls	Ruc	p53	K-Ras
1	194 ± 274	19,319 ± 310	480 ± 485
2	9 ± 13	9,830 ± 1606	1,064 ± 576
3	8 ± 11	5,236 ± 1380	445 ± 199
4	38 ± 53	3,187 ± 1093	477 ± 674
5	14 ± 19	11,908 ± 118	795 ± 447
6	31 ± 44	5,390 ± 1678	823 ± 112
7	76 ± 107	22,526 ± 8202	1,909 ± 103
8	29 ± 41	15,338 ± 274	943 ± 194
9	10 ± 14	12,282 ± 865	1,162 ± 445
10	9 ± 13	11,130 ± 1199	1,109 ± 38
Head and Neck Cancer			
11	0 ± 0	10,904 ± 4394	508 ± 117
12	0 ± 0	**31,593 ± 7200**	738 ± 460
13	0 ± 0	12,367 ± 1221	840 ± 486
14	13 ± 18	14,705 ± 274	1,012 ± 296
15	33 ± 46	**31,733 ± 274**	1,189 ± 654
16	121 ± 171	4,828 ± 274	621 ± 490
17	0 ± 0	8,517 ± 274	1,160 ± 437
18	0 ± 0	19,240 ± 274	1,283 ± 700
19	0 ± 0	11,224 ± 274	1,517 ± 241
20	28 ± 39	7,322 ± 274	554 ± 258
Breast Cancer			
21	44 ± 62	13,211 ± 3251	960 ± 897
22	49 ± 13	18,814 ± 5440	696 ± 295
23	38 ± 53	14,598 ± 2145	608 ± 81
24	77 ± 19	11,587 ± 3478	1,655 ± 493
25	17 ± 23	19,954 ± 1353	532 ± 39
26	25 ± 35	9, 538 ± 1933	195 ± 270
27	10 ± 13	7,815 ± 4052	**2,561 ± 1027**
28	21 ± 30	15,607 ± 1358	308 ± 166
29	0 ± 0	18,058 ± 4848	160 ± 56
30	**245 ± 339**	25,479 ± 150	1,919 ± 826
Colon			
31	4 ± 6	6,656 ± 1482	1204 ± 274
32	40 ± 57	20,928 ± 3785	**4,293 ± 1039**
33	42 ± 59	**34,703 ± 15,326**	1,472 ± 163
34	51 ± 71	**300,943 ± 104,539**	**6,439 ± 2953**
35	35 ± 49	5,670 ± 1321	**3,306 ± 851**
36	44 ± 62	6,516 ± 827	695 ± 520

[a]Values are averages plus or minus the standard deviation derived from two experiments.
[b]Numbers in bold are statistically significant: greater than the average plus 3 standard deviations of the 10 control sera.

Table 1. Mean and Standard Deviations of Sera Reactivity (cont.)

Controls	Smad4	β-CAT-Δ-1	c-Myc
1	10,582 ± 5262	269 ± 28	4752 ± 547
2	3575 ± 1814	835 ± 35	2913 ± 533
3	1773 ± 1857	211 ± 127	2006 ± 564
4	1919 ± 2713	530 ± 199	1831 ± 333
5	6884 ± 4453	161 ± 119	3346 ± 359
6	1724 ± 2050	235 ± 21	2050 ± 271
7	6996± 733	259 ± 132	11,816 ± 147
8	8043 ± 742	445 ± 335	3475 ± 333
9	19,380 ± 5128	215± 129	3623 ± 39
10	4429 ± 3434	501 ± 50	5060 ± 2611
Head and Neck Cancer			
11	2721 ± 3847	196 ± 23	2193 ± 39
12	4822 ± 296	465 ± 179	3801 ± 904
13	1868 ± 2582	673 ± 596	4407 ± 1165
14	5666 ± 4639	195 ± 158	1837 ± 152
15	5264 ± 166	552 ± 137	4107 ± 751
16	980 ± 1387	279 ± 88	1974 ± 26
17	8396± 5668	336 ± 168	2958 ± 832
18	9485 ± 4154	327 ± 44	1814 ± 444
19	4454 ± 2143	410 ± 421	4370 ± 1504
20	2261 ± 1889	723 ± 240	2343 ± 76
Breast Cancer			
21	10,219 ± 2746	308 ± 223	5988 ± 58
22	**42,970 ± 12,016**	302 ± 30	5450 ± 1089
23	8484 ± 938	339 ± 309	4336± 982
24	17,297 ± 1560	**2363 ± 1072**	3431 ± 1180
25	10,184 ± 4864	772 ± 114	**15,650 ± 57**
26	5962 ± 2650	300 ± 253	1646± 609
27	20,628 ± 2366	426 ± 214	3524 ± 574
28	7380 ± 6163	284 ± 257	1579 ± 164
29	6,790 ± 3899	304 ± 388	2333 ± 669
30	9727 ± 2099	495 ± 444	3787 ± 319
Colon			
31	3252 ± 287	267 ± 142	1763 ± 45
32	5567 ± 3034	962 ± 71	6143 ± 537
33	10,830 ± 8142	716 ± 516	4906± 521
34	2610 ± 1710	992 ± 80	3789 ± 73
35	3860 ± 4552	477 ± 446	1772 ± 890
36	**37,344 ± 14,788**	371 ± 324	2395 ± 517

Table 2 Competition of antibody responses by unmodified antigens[a]

Antigen/sera	Control	p53	K-Ras	Smad4	β-CAT-Δ1	c-Myc
p53/12	21% ± 29%	32% ± 7%				
p53/15	20% ± 28%	60% ± 16%				
p53/33	7% ± 9%	88% ± 1%				
p53/34	11% ± 9%	72% ± 20%				
K-Ras/27	5% ± 6%		91% ± 3%			
K-Ras/32	25% ± 4%		82% ± 26%			
K-Ras/34	4% ± 5%		0% ± 0%			
K-Ras/35	16% ± 23%		100% ± 0%			
Smad4/22	4% ± 6%			92% ± 1%		
Smad4/36	0% ± 0%			93% ± 1%		
β-catenin-Δ1/24	23% ± 33%				96% ± 6%	
c-Myc/25	0% ± 0%					22% ± 2%

[a]Sera (1 μl), buffer and 5 μg competitor were incubated together for 60 min before adding the fusion extracts and protein A/G beads for an additional 60 minutes and processed. Background light units (beads plus extract but no sera) were subtracted before calculating percent competition. The first column identifies the antigen-sera combination tested. The other columns give the amount of competition obtained for each competitor antigen. All competitors, including the control (SPEC2), are MBP fusion proteins. Values are the averages plus or minus the standard deviation derived from two independent experiments.

Table 3. Amount of protein (IgG) bound to A/G bead (mg/1ml) from different sera used in this study[a]

Controls	Amount IgG (μg/1μl)[b]
1	7.3
2	4.7
3	4.5
4	7.1
5	4.7
6	5.7
7	5.6
8	6.0
9	4.3
10	5.3
Head and Neck Cancer	
11	4.7
12	2.9
13	3.7
14	2.0
15	6.4
16	5.0
17	3.5
18	2.5
19	4.1
20	2.1
Breast Cancer	
21	5.2
22	4.4
23	4.0
24	3.6
25	5.4
26	3.8
27	5.2
28	4.3
29	4.2
30	4.6
Colon	
31	3.9
32	3.7
33	2.7
34	3.3
35	2.9
36	2.7

[a]Sera (2 ml), protein A/G beads and buffer were mixed together, incubated for 60 minutes and the beads washed as described for performing the immunoprecipitation. Bound protein was eluted with 0.1 M glycine, pH 2.3. The amount of protein in the eluant was measured using the BCA Protein Assay kit (Pierce Biotechnology Inc.).
[b]Values are from a single determination.
