# Supplementary material for: A simplified immunoprecipitation method for quantitatively measuring antibody responses in clinical sera samples by using mammalian-produced Renilla luciferase-antigen fusion proteins
Source: BMC Biotechnol. 2005 Aug 18;5:22. doi: 10.1186/1472-6750-5-22 (PMC1208859; doi:10.1186/1472-6750-5-22)
Supplement: Additional File 2 — Table 2. Competition of antibody responses by unmodified antigens [file 1472-6750-5-22-S2.pdf]

Supplementary Table 2 Competition of antibody responses by unmodified antigens [a]

Antigen/sera	Control	p53	K-Ras	Smad4	β-CAT-Δ1	c-Myc
p53/12	21% ± 29%	32% ± 7%				
p53/15	20% ± 28%	60% ± 16%				
p53/33	7% ± 9%	88% ± 1%				
p53/34	11% ± 9%	72% ± 20%				
K-Ras/27	5% ± 6%		91% ± 3%			
K-Ras/32	25% ± 4%		82% ± 26%			
K-Ras/34	4% ± 5%		0% ± 0%			
K-Ras/35	16% ± 23%		100% ± 0%			
Smad4/22	4% ± 6%			92% ± 1%		
Smad4/36	0% ± 0%			93% ± 1%		
β-catenin-Δ1/24	23% ± 33%				96% ± 6%	
c-Myc/25	0% ± 0%					22% ± 2%

[a] Sera (1 μl), buffer and 5 μg competitor were incubated together for 60 min before adding the fusion extracts and protein A/G beads for an additional 60 minutes and processed. Background light units (beads plus extract but no sera) were subtracted before calculating percent competition. The first column identifies the antigen-sera combination tested. The other columns give the amount of competition obtained for each competitor antigen. All competitors, including the control (SPEC2), are MBP fusion proteins. Values are the averages plus or minus the standard deviation derived from two independent experiments.
